# Medicine and the State

**Published:** 1911-07-15

**Authors:** 


### MEDICINE AND THE STATE.

### FROM A PROFESSIONAL CORRESPONDENT.

"What then are the qualities of a doctor who respects his profession? He must win the world's esteem by the profundity of his knowledge, the wide range of his experience, his integrity, and a blameless life. He is no distinguisher of persons; he must listen with sympathy to the history of their ills, go at once when they require him. He must inspire them with confidence, a potent factor in recovery. Fully appreciating their suffering, he must with perseverance study the cause and progress of their disease, never ruffled by unforeseen developments, never ashamed of asking advice from his colleagues. The ideal doctor is he, therefore, who, after striving might and main against the disease, is modest in success, though gratified, and where death ensues he can at least console himself with the thought that he has mitigated suffering."

This passage from Hippocrates will at least show that from the earliest times an ideal was set before the medical profession which, in the opinion of its author, raised the doctor to the level of a god. This ideal, consciously or unconsciously, has counted for much in elevating the medical profession as a whole, and it has acquired the reverence and respect inspired by an institution, anciently established and nobly perpetuated.

But nothing seems free from the vandalism of an age which delights to shatter one by one the idols of former times. There is now but little perception of the true inwardness of things, and it is becoming an anachronism to worship anything whose price is not written large upon it. Value is nothing, price is everything. Consequently it is scarcely surprising that a decade which sees an attempt to introduce payment of members of Parliament should also suffer a movement to turn the medical profession into a body of pettifogging hucksters, haggling over the prices of draughts and pills. If the State proposes to initiate a species of insurance which comprises a medical attendance without considering the opinion of the medical profession, it is time that the medical profession agitated for reforms which are as important as State insurance, if not quite so demagogic in conception.

Before any decision or agreement can be arrived at in the matter of insurance, it is imperative to consider from the individual standpoint the cost of a medical degree. Few men can obtain this without the expenditure of some hundreds of pounds, and the return that they get on their money often does not amount to one per cent. In a State in which the enlightenment of its Government has become a byword this seems a procedure that is anachronistic in the extreme. A fixed income should be guaranteed to each man on taking his degree, or hospitals and Universities should be subsidised by Government with a liberality sufficient to reduce the fees of the medical student to a minimum.

Or, again, it would be perhaps better to limit by State decree the number of doctors practising. In this case the existing doctors would have to be paid by the State, encouragement being given to research by a bonus system or some such State device. At any rate, either the medical profession must be left entirely free to arrange its own matters whether they appertain to State insurance, choice of a doctor, or internal politics, or else there must be an inquiry into the conditions of the profession as a whole and a suitable Parliamentary investigation. Owing perhaps to the hysterical use of the unwritten law there is a tendency at present to attack everything which has not yet received an exact definition, and the position of the medical profession, resting as it does on tradition and not on a written constitution, is obviously food for powder. Nevertheless, there is no body of men more deserving of conciliatory treatment. It is more than a mere question of insurance that is at issue. But the medical profession is not to be rudely handled and lightly set aside. It is unfortunately a sign of our modern statesmen that they appreciate only the sounds of words, and deceive themselves in thinking that a deputation of doctors can be treated no better than a body of school children asking for jam.

The ideal of Hippocrates is still the ideal of today, and a body of men such as this develops as it were unaided, and advances along a line of development chosen by itself. In a certain sense it may be said that nearly all the best work is unconscious. Certain changes in the development of a body of individuals or of an individual are not consciously premeditated but take place according to the need of circumstances. Consequently if Mr. Lloyd George's Bill is wrecked owing to the general opposition of the medical profession it will simply be an instance of a premature attempt to direct on conventional lines a body of men who have hitherto been permitted to arrange for their own salvation. At any rate, one thing is certain—no amount of quasi-legislative persecution is likely to change the attitude of the medical profession to the public in general, for whom they will continue to be a body of thoughtful men, actuated, with very few exceptions, by the noblest of motives—the hope that they can contribute to the mitigation of suffering.

